# Potassium Channel Antagonists 4-Aminopyridine and the T-Butyl Carbamate Derivative of 4-Aminopyridine Improve Hind Limb Function in Chronically Non-Ambulatory Dogs; A Blinded, Placebo-Controlled Trial

**DOI:** 10.1371/journal.pone.0116139

**Published:** 2014-12-31

**Authors:** Ji-Hey Lim, Audrey C. Muguet-Chanoit, Daniel T. Smith, Eric Laber, Natasha J. Olby

**Affiliations:** 1 Department of Clinical Sciences, College of Veterinary Medicine, North Carolina State University, Raleigh, North Carolina, United States of America; 2 Department Industrial and Physical Pharmacy, Purdue University, West Lafayette, Indiana, United States of America; 3 Center for Comparative Medicine and Translational Research, North Carolina State University, Raleigh, North Carolina, United States of America; 4 Department of Statistics, North Carolina State University, Raleigh, North Carolina, United States of America; Rutgers-Robert Wood Johnson Medical School, United States of America

## Abstract

4-Aminopyridine (4-AP) blocks voltage gated potassium channels, restoring conduction to demyelinated axons and improving function in demyelinating conditions, but its use is associated with adverse effects and benefit in spinal cord injury is limited. Derivatives of 4-AP have been developed to improve clinical efficacy while reducing toxicity. We compared the therapeutic effects of orally administered 4-AP and its t-butyl carbamate derivative (t-butyl) with placebo in dogs that had suffered an acute spinal cord injury that left them chronically paralyzed. Nineteen dogs were entered into the trial, conducted in two-week treatment blocks starting with placebo, followed by random assignment to 4-AP or t-butyl, a washout and then the opposite medication followed by placebo. Investigators and owners were blinded to treatment group. Primary outcome measures included open field gait score (OFS), and treadmill based stepping score and regularity index, with additional secondary measures also considered. Thirteen of 19 dogs completed the protocol. Two were euthanized due to unrelated heath problems, two developed side effects and two were unable to complete for unrelated reasons. Dogs showed significant improvement in supported stepping score (from 17.39 to 37.24% with 4-AP; 16.85 to 29.18% with t-butyl p<0.0001) and OFS (from 3.63 to 4.73 with 4-AP; 3.78 to 4.45 with t-butyl, p = 0.005). Response was individually variable and most dramatic in three dogs that were able to walk without support with treatment. No significant difference was found between 4-AP and t-butyl. No adverse effects were reported with t-butyl but gastrointestinal upset and seizures were observed in two dogs with 4-AP. In conclusion, both 4-AP and t-butyl significantly improved supported stepping ability in dogs with chronic spinal cord injury with no adverse effects noted with t-butyl. Drug response varied widely between individuals, highlighting the need to understand the factors that influence canine and human patients' response to therapy.

## Introduction

Severe traumatic spinal cord injury results in devastating destruction of spinal cord parenchyma [1]. However, over recent decades a body of evidence has emerged that axonal connections can be maintained across lesions, particularly in a sub pial location [1]. Demyelination of these axons due to oligodendrocyte death results in conduction block, but offers a potential therapeutic target. The presence of demyelinating lesions has been reported in experimental models of spinal cord injury [2], [3], and to a lesser extent in humans [1], [4], [5], [6] and dogs [7], [8] with naturally occurring injuries, although the clinical relevance of this pathology in humans remains controversial [9]. There has been interest in targeting these demyelinated axons using cellular replacement strategies [10], [11], [12] and using drugs, of which the potassium channel antagonist, 4-aminopyridine (4-AP) has proven the most promising.

4-Aminopyridine blocks rapidly activating voltage gated potassium channels [13] and was first noted to prolong the action potential and restore conduction to demyelinated axons in peripheral nerves [14]. It also enhances synaptic transmission [15] and contractile strength of muscle [16], leading to its early use in the treatment of neuromuscular disease. Its efficacy at restoring conduction across lesions in the acutely injured spinal cord in both the acute and chronic injury phases was established in *ex* and *in vivo* experimental models of spinal cord injury [17], [18], [19]. These experimental findings were translated successfully to clinical use for multiple sclerosis, a disease characterized by demyelination [20], [21], [22]. Efficacy has also been shown in humans with chronic spinal cord injuries in small clinical trials [23], [24], [25], but recently completed phase 3 trials failed to show benefit [26], and use of 4-AP is limited by significant adverse effects such as tremors and seizures at clinically effective doses [23], [27]. These clinical trials suggest that demyelinated axons may not provide a clinically relevant therapeutic opportunity in spinal cord injury. However, given the variability in the extent and nature of pathology in patients with spinal cord injury [1], [4], [5], [6], it is still possible that potassium channel blockade could be of benefit to a patient subset, particularly if a more safe and effective drug was available.

Derivatives of 4-AP have been developed with the aim of improving clinical efficacy while reducing toxicity. Three carbamate derivatives of 4-AP showed efficacy in restoring conduction to stretch-injured guinea pig white matter strips isolated from the spinal cord and evaluated *ex vivo* in a recording chamber, and in an *in vivo* model of spinal cord injury [28], [29], [30]. Two of the derivatives were effective at similar recording chamber fluid concentration ranges to 4-AP *ex vivo* while the t-butyl derivative was 100 fold more potent [29]. Moreover, the electrophysiological characteristics of conduction restored to axons by the derivatives were more similar to those of normal axons than 4-AP treated axons [30]. More recently, we reported the pharmacokinetics and safety of all three derivatives (methy-, ethyl-, and t-butyl carbamate derivatives) in normal dogs [31]. All of the derivatives had an excellent safety profile when administered at doses that achieved potentially therapeutic blood and cerebrospinal fluid (CSF) levels. A small dose response study of the t-butyl derivative (t-butyl) in dogs showed efficacy in the 0.1 to 1 mg/kg dose range [32]. We therefore wished to investigate the safety and efficacy of t-butyl more rigorously in a chronic model of acute spinal cord injury and to compare its performance with 4-AP.

Dogs with naturally occurring spinal cord injuries resulting from acute disc herniations or external trauma causing variable combinations of contusion, compression and laceration model human spinal cord injury. They have been identified as a clinically relevant model to translate experimental results from small animal models to clinical use in humans [33], [34], [35]. Indeed, 4-AP was tested in phase 1 trials in this population of dogs prior to moving to human testing [23]. In this blinded, placebo-controlled, cross-over trial, we evaluated the efficacy and adverse effects of 4AP and t-butyl in dogs that had suffered acute spinal cord injuries, leaving them chronically non-ambulatory.

## Materials and Methods

### Animals

Chronically non-ambulatory paraparetic or paraplegic client-owned dogs were recruited to the study. In order to be included, dogs had to have a naturally occurring severe, acute thoracolumbar spinal cord injury involving the third thoracic to third lumbar spinal cord segments a minimum of three months previously. The injury had to result from either an acute intervertebral disc herniation or a traumatic event. These were diagnosed at the time of injury using CT or MRI, with appropriate surgical treatment provided at that time; these details were established by review of the dogs' medical records. At the time of injury, they were assessed to have functionally complete lesions (paraplegia with absent pain perception) [36] from which they had made an incomplete recovery leaving them non-ambulatory with neurogenic urinary and fecal incontinence. All dogs were required to have stable neurological function for a minimum of one month before entry into the study. Dogs were excluded if they had a systemic condition that might prevent use of the drugs (for example, a seizure disorder), or an orthopedic or secondary neurologic condition (such as severe muscle contractures) that might alter their response to the drugs and gait assessment. If a dog had a urinary tract infection, it was treated before entry into the study. Dogs were also excluded if they did not tolerate walking on the treadmill, or behaved aggressively to handlers during the initial evaluation.

#### Ethics statement

The protocol was reviewed and approved by North Carolina State University (NCSU) Institutional Animal Care and Use Committee (#09-068-O). Owners of dogs were provided with written informed consent prior to participation and once entered into the trial, owners were asked to keep their pet's exercise routine consistent for the duration of the study.

### Study design

**Figure 1:**
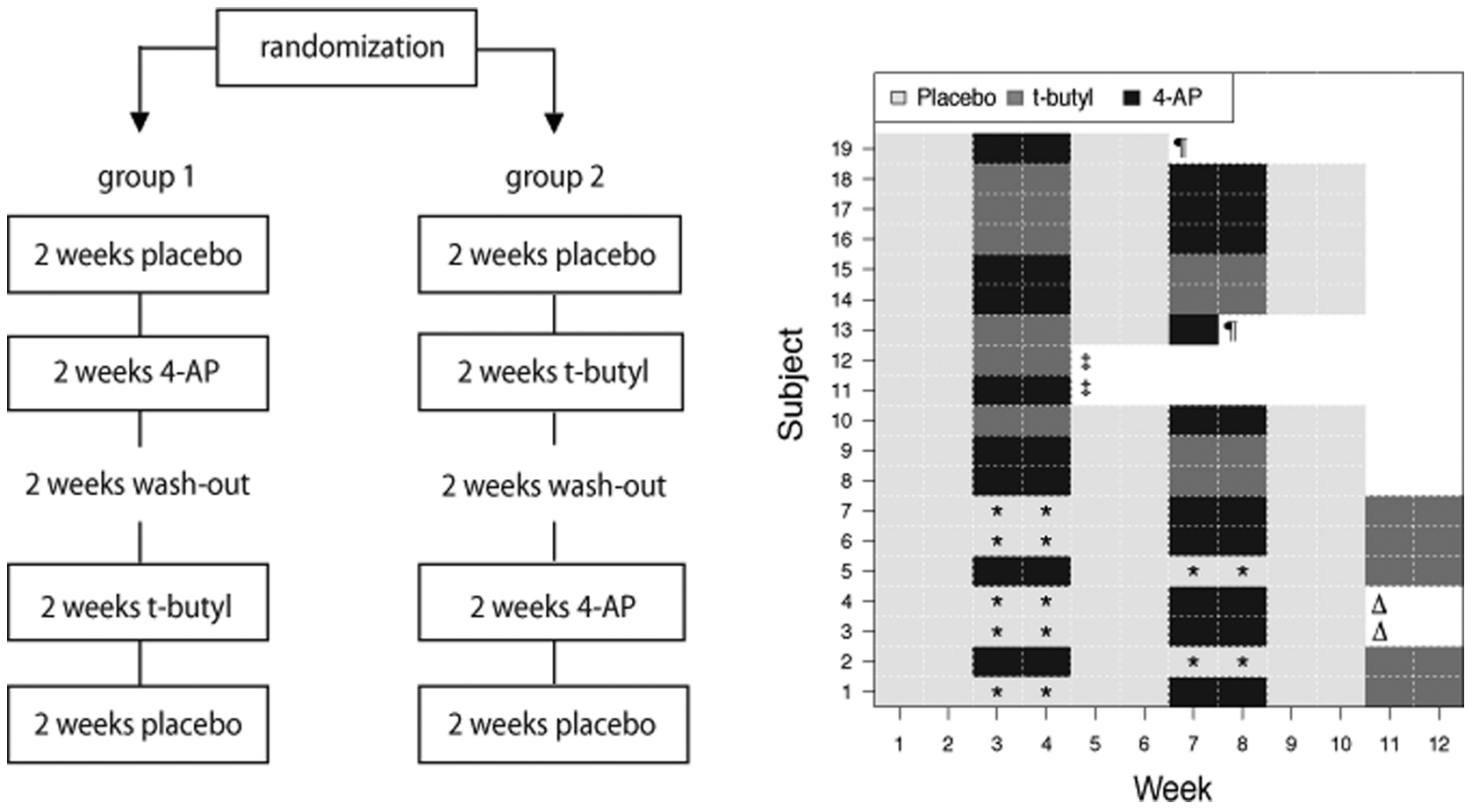
Study design and patient treatment allocation. A: Diagram of the study design. B: Treatment assignments for each subject by week. Boxes indicated by an asterisk (*) represent sub-therapeutic dosing with t-butyl. These and the preceding two-week treatment periods were removed from statistical analysis. Symbols indicate dogs that did not complete the trial; Δ: owners unable to continue; ‡: euthanasia; ¶: discontinuation due to adverse effects. Dog 13 received just 2 doses of 4-AP prior to development of seizures. Dog 19 received 10 doses of 4-AP prior to developing gastrointestinal signs.

This trial was a blinded, placebo-controlled, cross over study that was designed according to the Guidelines for the Conduct of SCI trials [37]. Dogs were randomized to one of two treatment groups by the NCSU College of Veterinary Medicine pharmacy (www.random.org/integers/). The design of the study is illustrated in Fig. 1. In weeks 1 and 2, all dogs received placebo to establish their baseline function. In weeks 3 and 4, dogs in group 1 were treated with 4-AP and dogs in group 2 received t-butyl. Weeks 5 and 6 were a washout period and in weeks 7 and 8 the treatments were crossed over. Finally in weeks 9 and 10 they received placebo. Owners administered all treatments orally 3 times a day and were blinded to the treatment (4-AP, t-butyl or placebo) being administered throughout the study. Two separate bottles of medication were dispensed every 2 weeks; one was given in the morning and at night, and the other in the middle of the day. Both bottles contained identical doses of t-butyl when t-butyl was being administered as it was dosed TID. In contrast, 4-AP was administered twice daily, so one bottle contained placebo (identical in appearance to the 4-AP) that was given in the middle of the day during 4-AP treatment periods. All capsules (4-AP, t-butyl and placebo) were compounded by the NCSU College of Veterinary Medicine pharmacy to appear identical. The investigators were also blinded to the treatment (4-AP or t-butyl) being administered but not to the week of the study during data collection.

Several different outcome measures were obtained on a weekly basis (Table 1) in order to obtain objective continuous measurements of function (treadmill based scoring of stepping and coordination), and to quantify less controlled movements more representative of what the dog was capable of when walking in the open field (open field score, OFS) (S1 Table). Blinded owner assessments of changes in a number of different parameters were also scored (S1 Fig.), as well as other aspects of the neurological examination performed by the investigators (Tables 1 and 2). The primary outcome measurements identified during study design were the treadmill based scores and the OFS. The other parameters were considered secondary outcome measurements.

**Table 1:** Outcome measures.

Outcome measure	Data type
***Primary outcomes***	
Open field score of gait (OFS)	Ordinal
Stepping score: treadmill based score with & without support	Continuous
Regularity index: treadmill based coordination score with & without support	Continuous
***Secondary outcomes***	
Voluntary tail wag	Owner assessment: categorical
Urination	Owner assessment: categorical
Walking with support	Owner assessment: categorical
Independent walking	Owner assessment: categorical
Pain perception	Ordinal
Proprioceptive placing	Ordinal
Segmental reflexes; patellar reflex & hind limb withdrawal reflex	Ordinal
Cutaneous trunci reflex	Continuous

**Table 2:** Ordinal testing scales.

Parameter	Scoring per hind limb	Total normal score
	0:absent	
**Proprioceptive placing**	1:reduced	4
	2: normal	
**Pain perception**	0:absent	
Tail	1:reduced	6
Hind limbs	2: normal	
**Spinal reflexes**	0:absent	
Patellar reflex	1:reduced	4
Withdrawal reflex	2: normal	
	3: hyper-reflexive	

In order to determine the number of cases needed to have adequate statistical power for this trial, we first considered data collected in a previous study on the long-term outcome of dogs with severe, acute spinal cord injuries [36]. In this study 18 dogs that suffered a severe spinal cord injury due to an acute disc herniation failed to recover pain perception or motor function by 1 month after injury. Eight of these dogs showed delayed recovery of ambulation in spite of lack of recovery of pain perception. However, all 8 dogs showed preliminary signs of recovery of motor function and a voluntary tail wag by 3 months after injury. In the same study, none of the dogs that suffered a severe traumatic spinal cord injury with displaced fractures recovered motor function. By limiting dogs entering this trial to those that show no or limited recovery of function that has plateaued for 1 month (with no voluntary tail wag) by 3 months after injury, we can anticipate that these dogs are extremely unlikely to recover in the longer term [36], [38]. Preliminary data from the dose escalation study was used to perform a power analysis for this study, but the dataset was limited, and the underlying cause of paralysis was not well defined in all dogs. In that preliminary dataset, 2 of 7 dogs recovered independent ambulation with t-butyl and the mean improvement in score was 17%. Power calculations showed that a group size of 10 dogs would have a 93% chance of detecting a 17% improvement. However, we decided to take a more conservative approach to the study design, and predicted an 11% improvement in open field score (1.5 points) in which case 20 dogs would give a power of 92%.

### Drug dose titration

Potential study participants were hospitalized for 3 to 5 days for titration of 4-AP doses in order to optimize beneficial effects while minimizing the known adverse effects. Dogs were acclimatized to the treadmill daily during hospitalization. Titration of t-butyl was not undertaken based on the safety of this drug established in previous work [31] and a dose rate of 1 mg/kg three times a day was used based on a small dose escalation study in dogs [32]. The starting dose of 4-AP was between 0.5 and 0.75 mg/kg orally depending on dog size. Dogs were monitored for drug response by walking them outside every 2 hours for 6 hours. In previous work, peak plasma levels were seen at around 2–3 hours after oral administration [31] with clinical effects lasting approximately 5 to 8 hours. Dogs were monitored continuously for the presence of adverse effects including anxiety, hyperesthesia, tremors or seizures for 6 hours after dosing. If there were no adverse effects, the dose was increased to between 0.75 and 1.2 mg/kg on subsequent days and this dose, if well tolerated by the patient, was used for the trial. If adverse effects were seen at this dose, or no further benefit was identified, the initial dose was used, administered twice daily.

### Data collection

Dogs were evaluated once a week throughout the trial period by two investigators. One investigator ensured that the video camera was set up and recording correctly, operated the treadmill and restrained the patient as needed, while the other performed the examinations and supported the dog when needed for gait evaluation. On the morning of evaluation, the medication (drug or placebo, identical in appearance) was given when the dog arrived at the clinic and the evaluation was performed 2 hours later. A routine physical and neurological examination and detailed gait assessment were performed at each evaluation. The neurological examination and gait assessment were videotaped; each video clip was identified with randomly generated numbers (www.random.org) and was downloaded onto a computer immediately after the evaluation for scoring at a later date, thus ensuring that observers were blinded to treatment period when scoring.

The neurological examination included testing for the presence of pain perception in the pelvic limbs and tail by applying heavy pressure using hemostatic forceps, testing proprioceptive placing in the pelvic limbs by placing the paw onto its dorsal surface while supporting the dog in a standing position, and testing the patella and withdrawal reflexes [39]. These parameters were scored using standard ordinal scales at the time of testing (Table 2) [40], [41]. The caudal level of the cutaneous trunci muscle (CTM) reflex was also recorded at each visit [42].

For the gait assessment, dogs were videotaped walking on a treadmill (Ironman Inspire) and on a 15-foot long nonslip mat both with and without support of their hind-quarters in order to document both weight bearing and non-weight bearing hind limb stepping using methods developed in our laboratory [43], [44]. It was noted that some dogs step more with tail support and others with sling support (walkabout). In order to maximize stepping, the optimal method of support was first established for each dog and then adopted throughout the trial. For all but one dog (dog 17), tail support was the optimal method. The video camera was set up on a tripod to capture movements of all 4 limbs, and the treadmill speed adjusted to a comfortable walking speed for the dog. Videotape of the dogs' supported and unsupported gait walking for a minimum of 45 seconds when unsupported and 75 seconds when supported was obtained based on data that demonstrated that scoring 50 continuous step cycles provides reliable and representative data [43]. Dogs were then videotaped walking along a flat non-slip mat to generate an OFS as previously described [44]. Briefly, walking over a distance of 15 feet was recorded twice each from the dogs' right and left sides and behind both with and without support. Owners completed a take-home questionnaire each week (Fig. S1).

### Scoring

The video clips of each evaluation, identified by random numbers, were scored at the study end in order to ensure blinding of the observers (J-HL and NJO). The OFS was generated using the previously reported 14-point scale, modified to exclude assessment of pain perception, which was scored separately [33], [44] (S1 Table). The relative frequency of weight bearing and non-weight bearing hind limb stepping (“stepping score” generated from the number of hind limb steps divided by the number of fore limb steps) and the number of coordinated step cycles (“regulatory index” generated from exclusive use of normal step sequence patterns during uninterrupted stepping) were documented [43], [45]. The mean of both investigators' scores for each videotape was used for statistical analysis. Owner questionnaires were evaluated to determine whether the owner observed deterioration (D), no change (N), or improvement (I) in each parameter assessed (Fig. S1). These included tail wagging, bladder function, motor function without support and motor function with support. The DNI categories were used for statistical analysis with D classified as −1, N = 0 and I = +1.

### Data analysis

We considered the effect of treatment on outcomes listed in Table 1. To account for individual subject effects a model was derived that considered all outcomes under treatment relative to the outcome under the 2-week period immediately preceding treatment. The model also allowed testing for carryover effect by comparing outcomes in the three untreated periods (weeks 1/2, 5/6 and 9/10). A full mathematical description of the derivation of the model is provided in the supplementary materials (S1 Text). Data was fit to the model using maximum likelihood and the model was used to test the null hypothesis of no treatment effect for each drug separately (control period (2 week period immediately preceding treatment period) scores compared to drug treatment scores) and no difference in treatment effect between drugs. P values were adjusted for multiplicity via the Bonferroni correction for the 7 parameters that were tested: a 0.05 significance level becomes 0.05/7 = 0.007. All analyses were performed using the R programming language (***cran*** *.us.r-project.org/*).

## Results

Nineteen dogs were enrolled in the study. Details of the dogs' signalment, cause and level of injury, treatment at the time of injury, duration of signs prior to entrance into the study, and individual drug doses are provided in Table 3. Thirteen of the 19 dogs successfully completed the full protocol. Of the remaining 6 dogs, 2 (nos. 11 and 12) were euthanized due to the development of health problems unrelated to the study, 2 (nos. 3 and 4) were unable to complete the study due to changed circumstances of the owners making travel impossible and 2 (nos. 13 and 19) were removed due to the development of possible adverse effects (Fig. 1). One of these dogs (no. 13) developed tremors, anxiety and seizures 2 days after starting 4-AP therapy at 1.01 mg/kg dose. The signs were responsive to intravenous diazepam, no further doses were administered and the dog was removed from the trial. The dog was later diagnosed with primary epilepsy and so was likely predisposed to develop seizures. The other dog (no. 19) developed severe vomiting and diarrhea after 10 days of 4-AP at 0.7 mg/kg. Dosing with 4-AP was discontinued immediately, but given the non-specificity of vomiting and diarrhea, and the late appearance of the signs during the drug dosing period, the dog was evaluated twice more during the wash out period. However, gastrointestinal signs continued to wax and wane and the dog was removed from the study. Data from the washout period was not used in further analysis. This dog was subsequently diagnosed with inflammatory bowel disease and it is unclear whether the signs were related to the drug. Neither dog had shown any adverse effect during dose titration. No adverse effects were noted with t-butyl. Seven of 19 patients received t-butyl initially at a sub-therapeutic dose. Five of these dogs were entered into another round of testing and received t-butyl at 1 mg/kg after completion of weeks 9 and 10 (Fig. 1). Two of the dogs dropped out of the trial prior to receiving the optimal dose of t-butyl due to changes in owner circumstances making travel impossible (see above), and their data as regards to the suboptimal dose and the 2 weeks of placebo preceding this dose were excluded from analysis (Fig. 1 and S1 Dataset).

**Table 3:** Patient signalment.

No	Breed	Age* (m)	Wt (kg)	Gender	Cause of injury	Lesions	Duration of signs* (m)	4AP (mg/kg)	t-butyl (mg/kg)	Surgery at the time of injury
										
1	Dachshund	156	5.6	MC	IVDD	T13-L1	108	0.83	1	no
2	Cocker spaniel	48	15	MC	IVDD	T12-L1	4	1.2	1	yes
3	Dachshund	83	7.6	MC	IVDD	T12-13	9	0.96	0.12	yes
4	Doberman	79	25	FS	T-SCI	T13-L1	6	1	0.2	yes
5	Dachshund	10	6.4	MC	T-SCI	T13-L1	4	0.97	1	no
6	Pug	61	8.2	M	T-SCI	T13	24	1.12	1	yes
7	Shih Tzu	93	6.2	M	IVDD	T13-L1	44	1.2	1	yes
8	Dachshund	99	7.3	MC	IVDD	T12-13	10	1.02	0.7	yes
9	Schnauzer	50	9.4	FS	IVDD	L1-L2	17	1.06	1	no
10	Dachshund	72	6.3	MC	IVDD	L2-L4	24	1	1	yes
11	Doberman	25	36	MC	IVDD	L1-L2	8	0.97	1	yes
12	English springer spaniel	120	26	MC	IVDD	T12-13	6	0.98	1	yes
13	Labrador retriever	30	27	MC	T-SCI	L3	12	1.01	1	yes
14	Corgi pembroke	72	18	M	IVDD	L1-L2	6	0.92	1	yes
15	Pekinese	72	6.6	MC	IVDD	L2-L3	6	0.94	1	yes
16	Bichon	92	11.2	FS	IVDD	T11-T12	7	0.89	1	yes
17	Schnauzer	66	8.8	MC	T-SCI	T11-T12	24	0.75	1	no
18	Dachshund	44	5.2	M	IVDD	L1-L2	4	0.76	1	yes
19	Dachshund	120	5.2	FS	IVDD	T12-13, L2-3	4	0.7	1	yes

Wt: weight; MC: male castrated; FS: female spayed; M: male; IVDD:intervertebral disc disease (type I); T-SCI: traumatic spinal cord injury; m:months;

*: at the time of enrollmen.

The hind limb neurological function of these dogs at entry (mean of the first two weeks data) was extremely limited (S1 Dataset). None of the dogs could generate weight-bearing steps on the treadmill without support (stepping score and regularity index of 0). With support, their mean stepping score was 17.32% (range 0–63, median  = 11.00, sd = 19.29) and mean regularity index was 5.04% (range: 0–20.48, median  = 0, sd = 6.78). Seventeen dogs had absent pain perception in their tails or hind limbs (pain perception score of 0). Two had reduced pain perception in one hind limb only (pain perception score of 1). Their proprioceptive placing scores ranged from 0 to 1 (mean  = 0, median  = 0, sd = 0 (score 0 (n = 14), score 1 (n = 5)). Eight of the dogs had cranial migration of the caudal border of their cutaneous trunci reflex reflecting the spinal level of their injury and 11 dogs had a normal reflex, again reflecting the level of their injury. On evaluation of patellar reflexes, 10 of the dogs had hyper-reflexia and 9 dogs had a normal reflex. Withdrawal reflexes were normal in all dogs. None of the dogs could urinate voluntarily, all were being manually expressed by their owners.

There were no changes in the level of the cutaneous trunci reflex, or the ordinal scores for proprioceptive placing, patellar and withdrawal reflexes, or pain perception in any of the dogs throughout the study period. No further statistical analysis was performed on this data.

**Figure 2:**
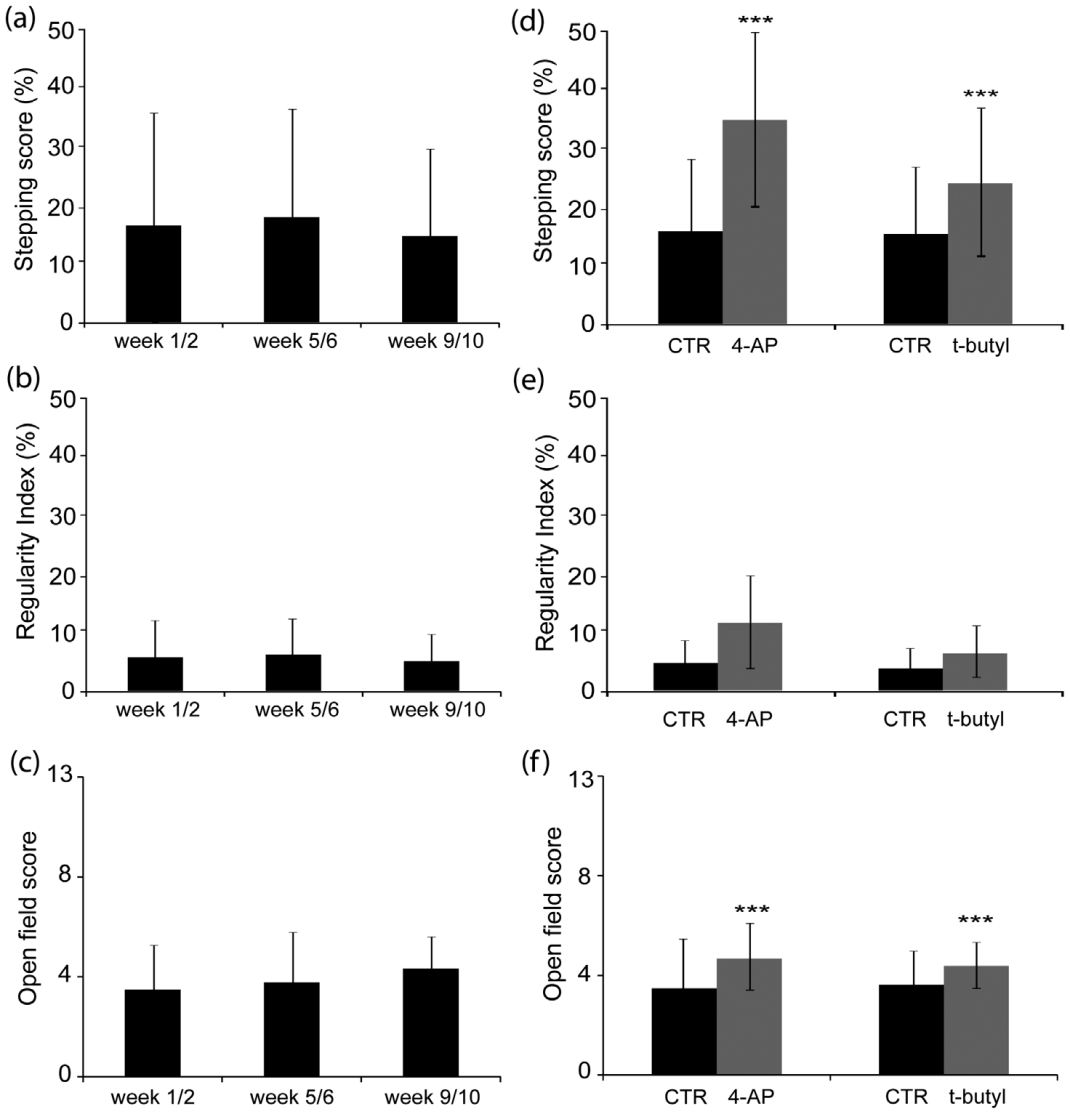
Results of the primary outcome measurements of gait. Mean supported stepping score (a), regulatory index (b) and OFS (c) during weeks 1–2, 5–6 and 9–10 did not change significantly (see p values for *ß* ^k^_1_ provided in Table 4), showing that there is no significant treatment effect carried over from the treatment periods. Mean supported stepping score (d), regulatory index (e) and OFS (f) for 4-AP and t-butyl treatment periods compared to the preceding 2 week period. Stepping score and OFS show significant improvement with treatment (***) (see p values for *ß* ^k^_0_ provided in Table 4); Data represents mean ± SD, CTR: the 2-week control period immediately preceding treatment.

Changes in the primary outcome measures are illustrated in Fig. 2 and all outcomes are listed in Table 4. Complete study data is provided in the supplementary materials (S1 Dataset). There was no significant change in any of the scores between the untreated weeks 1/2, 5/6, and 9/10, demonstrating that there was no treatment carry over once treatment with 4-AP or t-butyl was discontinued and no learning or training effect through the course of the trial (Fig. 2a-c and Table 4). Thus, each block of 4 weeks (control and treatment) could be considered as an independent block of data. Data were available from one 4-week block in all 6 dogs that did not complete the full protocol and were incorporated into the analysis (Fig. 1).

**Table 4:** Outcome data.

Outcome measure	Control weeks prior to 4-AP Mean (SD)	4-AP Mean (SD)	Control weeks prior to t-butyl Mean (SD)	t-butyl Mean (SD)	*ß* ^k^_0_	P value	*ß* ^k^_1_	P value	Ave. Imp.
OFS	**3.63** (2.10)	**4.73** (1.42)	**3.78** (1.57)	**4.45** (1.45)	0.62	0.005	0.15	0.31	0.66
SS + support	**17.39** (13.57)	**37.24** (20.94)	**16.85** (11.65)	**29.18** (20.28)	13.6	<0.0001	1.9	0.51	13.4
SS -support*	**2.51** (4.89)	**5.71** (13.72)	**0.98** (1.70)	**5.32** (13.93)					
RI + support	**4.95** (3.79)	**13.1** (11.95)	**3.98** (3.67)	**6.97** (8.83)	4.7	0.028	2.2	0.13	4.9
RI -support*	**0.79** (1.54)	**1.84** (4.99)	**0.36** (0.63)	**1.45** (4.07)					
HL DNI + support	NA	D:0 N:7 I:10	NA	D:0 N:6 I:9	0.17	0.035	0.17	0.13	0.66
HL DNI no support*	NA	D:0 N:11 I:6	NA	D:0 N:10 I:5	0.25	0.076	0.061	0.54	0.27
Tail wag DNI	NA	D:0 N:13 I:4	NA	D:0 N:12 I:3	0.055	0.29	0.002	0.94	0.0625
Urination DNI	NA	D:0 N:15 I:2	NA	D:0 N:15 I:0	0.059	0.18	0.059	0.18	0.063

Control weeks: 2 weeks period immediately preceding treatment with either 4-AP or t-butyl; OFS: open field score (0–13); SS: stepping score; RI: regularity index; DNI: decreased (−1), no change (0), improved (+1); HL: hind limb; *ß* ^k^_0_: treatment effect of 4-AP or t-butyl relative to control; *ß* ^k^_1_: relative treatment effects of 4-AP and t-butyl. Positive values of *ß* ^k^_1_ reflect outperformance by 4-AP, and negative values reflect t-butyl. The scores denoted with * were not changed by treatment, therefore *ß* ^k^_0_ and *ß* ^k^_1_ values could not be calculated.

There was a statistically significant improvement in supported stepping score and in the OFS with both 4-AP and t-butyl using correction of p values for multiple comparisons, and in the supported regulatory index and owner assessment of hind limb function when using a p value of 0.05 without adjustments for multiplicity (Table 4). There was no significant difference between the two treatments although the relative difference between 4-AP and t-butyl (*ß* ^k^_1_ : see S1 Text) was positive for most parameters, reflecting slightly better performance with 4-AP (Table 4). Closer evaluation of individual dogs revealed that 5 dogs showed no change in supported stepping, while the remaining 14 all showed an improvement in score, with a maximum change of 69.4% in one dog.

When unsupported on the treadmill, 16 dogs were still unable to generate any weight-bearing steps with either treatment; unsupported stepping and refractory index scores remained at or close to zero. However, 3 dogs did show a response with improvements in both the unsupported stepping score and regulatory index. These dogs changed from unable to generate unsupported weight bearing steps to independent stepping (S1 Video). Two of these dogs responded to t-butyl and 4-AP and 1 responded to 4-AP alone. These dogs simultaneously showed increases in their OFS and all 3 owners identified the improvements in their blinded assessments, suggesting that the improvement was not simply driven by movement of the treadmill belt and was perceived to be significant by owners.

Owner assessments of changes in voluntary tail wag and urination (including ease of bladder expression) did not change significantly over the treatment period. However 4 owners did note that voluntary tail wagging improved, and 2 owners reported that bladder expression was easier during treatment periods. Owners of 1 dog that failed to change in terms of hind limb motor function continued to use 4-AP in the long term due to the improved management of urination and the presence of a voluntary tail wag. Owners perceived recovery of tail wag as a very positive event in their daily interactions with their pet.

## Discussion

This clinical trial demonstrated that the potassium channel antagonists 4-AP and t-butyl both significantly improve supported hind limb stepping ability in chronically non-ambulatory dogs. This walking ability was measured in a blinded fashion using an ordinal scale that is representative of function when walking freely on a non-slip surface, and using continuous stepping and coordination scores when walking in a more controlled environment with weight support on a treadmill. In addition, owners blinded to treatment detected an improvement in motor function. Response to the drugs was extremely variable, ranging from no change in scores to a subset of 3 dogs that improved enough to take unsupported steps. These drugs represent a viable therapeutic option for a subset of dogs left chronically disabled by acute disc herniation or traumatic spinal cord injury. Moreover, this study highlights the need to investigate parameters that might predict patient response to these drugs, and to consider the possibility that individual patients, both canine and human, with spinal cord injury may show a dramatic improvement.

We performed a blinded, placebo-controlled cross over study in dogs with naturally occurring spinal cord injuries. Dogs with acute spinal cord injury due to acute intervertebral disc herniations have been used as a clinically relevant model to translate data generated in the controlled spinal cord injury models used in rodents to the more diverse and complex naturally occurring injury in humans [23], [38]. The majority of dogs used in this study had suffered acute intervertebral disc herniations (n = 14), while the remainder had suffered traumatic injuries. In dogs, there are 7 lumbar spinal cord segments and all dogs recruited to the study had lesions above the level of the lumbosacral intumescence, thus all had spastic paralysis by the time of presentation. The dogs presented with clinically complete lesions (no pain perception or motor to either hind limb or tail) at the time of injury and failed to recover motor function successfully, entering this clinical trial as chronically (at minimum 4 months) disabled dogs. At time of entry into the trial, seventeen of the dogs were comparable to human ASIA grade A, and 2 had limited sensation but no voluntary motor function, comparable to ASIA grade B. This was a unique canine patient population that modeled the chronically paralyzed human population both in terms of chronicity (a mean duration of paralysis of 17 months at time of trial entry), and in terms of mixed contusive and compressive lesions.

In dogs, scoring of motor and sensory function on a segmental basis as for the ASIA scale is challenging because they can't be asked to voluntarily contract individual muscle groups, or to respond to different types of sensory stimulation beyond stimulation of behaviorally recognizable pain perception. Outcome measures in our study included ordinal scales to assess gait, sensation and proprioception, complemented by continuous gait scores assessing stepping ability and coordination and owner generated assessments of changes in a number of functions at home. The ordinal scales have been validated by both our laboratory and other groups [33], [41], [44], but detect quite large changes in function only. The treadmill based stepping and coordination (regulatory index) scores allow more detailed evaluation of recovery of ambulation with the potential to discriminate between stepping through local circuits versus descending input, important to our understanding of the mechanism of improvement [43], [45]. Owner generated assessments allowed evaluation of the impact of the therapy on the patient at home over a longer period of time. The fact that improvements were detected in all 3 types of data (ordinal gait scales, continuous gait data and owner questionnaires), suggests our findings are robust and compare favorably with the recovery of coordination between forelimbs and hind limbs described in another trial in a similar population of dogs [38].

To overcome the natural variations in baseline that occur in these chronically paraparetic animals, we used a time-scale of 2 weeks so that a mean of 2 weekly sets of scores could be used, and we included blinded owner observations covering entire one-week periods. All dogs followed the sequence of placebo, randomized drug, washout, randomized drug and ‘placebo’ and our statistical model allows for patient specific effects (S1 Text). Our data demonstrated that there was no treatment carryover effect or learning effect (Fig. 2), thus, the effect of the drugs could be compared to the preceding control period, allowing us to use data from dogs that did not complete the entire protocol. Ultimately, we evaluated data from 17 subjects receiving 4-AP and 15 subjects receiving t-butyl; 6 dogs did not complete the trial, which may have impacted the study power. In addition, an error in t-butyl dosing resulted in 13 dogs receiving 4-AP first with only 6 dogs receiving t-butyl first, affecting the randomization.

4-Aminopyridine produces a dose dependent blockade of rapidly activating voltage gated potassium channels unmasked by demyelination when used in patients with spinal cord injury or multiple sclerosis [13], [46]. However, it has also been proposed that 4-AP might lead to clinical improvement by an increase in neurotransmitter release or an increase in the number of activated synaptic terminals [47]. In this study, significant improvement was shown in supported stepping scores and open field scores, which could be mediated by increased activity in the central pattern generators, descending motor control or a combination of both. Significant improvement in a kinematically generated measure of fore limb hind limb coordination has been reported in a study that investigated the effect of the transplantation of olfactory mucosa cells in a similar population of chronically disabled dogs [38]. In that study, there was no electrophysiological demonstration of restoration of conduction via long tracts or significant functional improvement in lateral stability of foot placement, which is controlled by descending (rubrospinal) pathways, leading to the proposal that the improvement was mediated via plasticity in propriospinal pathways. The current clinical trial was somewhat different to the transplantation trial in that treatment effect was an immediate pharmacologic effect that disappeared once the drug was withdrawn, suggesting plasticity was less important, but the mechanism of recovery is unclear. The observation by some clients of recovery of voluntary tail wagging in response to positive stimuli, is suggestive of some recovery of descending control. There was no change in the level of the cutaneous trunci reflex, proprioceptive placing or pain perception in any dog all of which would be mediated by the long sensory tracts. In an earlier tolerability study in a similar population of dogs (disabled due to SCI with chronicity from 3 months to 24 months), using a single IV or oral dose of 0.5–1 mg/kg 4-AP, improvements in stepping ability, the level of cutaneous trunci reflex, proprioceptive placing and pain perception were detected in some dogs [23]. The difference in our findings on sensory function could be related to peak blood levels attained following IV administration versus oral administration, which may also be reflected in the increased incidence and severity of adverse effects encountered in the previous study.

The failure of 4-AP to show benefit in three large phase 3 clinical trial in humans with spinal cord injury is disappointing [26]. The results of these 4-AP trials suggest that demyelination may not be clinically relevant in this human population. However, the results of our study, and positive results in smaller phase 2 human clinical trials [13], [23], [48], [49], [50] highlight the importance of variability in the patient population. While the dogs in this study had suffered from similar acute injuries, in similar regions of the spinal cord, and all presented with the same initial neurologic grade, their responses to 4-AP and t-butyl were variable. Overall there was a significant improvement in supported stepping ability and open field walking, but the magnitude of these changes was small. The impact of moving from a score of 3.6 to 4.7 on the open field score (the mean change for the entire group) reflects increased frequency of non-weight bearing limb movements (also captured on the doubling of the supported stepping scores) but not the ability to walk independently (S1 Table). It is notable however, that owners of dogs that did not show much motor response to these drugs opted to continue treatment with 4-AP (as the only drug consistently available) because of perceived benefits on bladder management and on tail wag. A closer evaluation of the data reveals that the majority of patients showed small benefits, 5 patients showed little to no response, and 3 patients showed a dramatic response, improving from non-ambulatory to independently ambulatory when on drug therapy (S1 Video). We propose that this variable response is influenced by many factors, central of which may be the underlying pathology, both in terms of presence and extent of demyelination, involvement of particular tracts, craniocaudal lesion extent, in addition to the proximity of the injury to central pattern generators, duration of paralysis, animal age [51], and pharmacogenomic factors. Closer investigation of these factors in a larger patient cohort, and the development of personalized therapies are likely to be of vital importance to optimizing treatment of people.

In the present study we demonstrated improvement in stepping ability using both 4-AP and t-butyl. We used careful dose titration of 4-AP for each patient, given the risk of adverse effects and also the observations that the optimal dose of 4-AP may not be the maximum tolerated dose. We did not perform this step for t-butyl, due to the lack of adverse effects in our pilot study. The findings of prior *ex vivo* and *in vivo* electrophysiological studies suggested that t-butyl was more potent, and restored more physiologically normal conduction to injured axons than 4-AP [28], [29], [30]. A tolerability and pharmacokinetic study compared three carbamate derivatives of 4-AP in dogs and demonstrated that t-butyl was more lipophilic than 4-AP, and was well absorbed orally, achieving potentially therapeutic levels in blood and CSF with no adverse effects [31]. Thus, this derivative was chosen for further study in clinical patients. The dose of t-butyl selected in this trial was chosen based on a previous small dose escalation study in clinical patients [32]; further optimization of the dosing of t-butyl may confer additional benefit. Detailed study of the mechanism of action of t-butyl and how it might differ to 4-AP has not been completed, but given the significant improvement of stepping and the lack of adverse effects noted in dogs in this trial, further study of its mechanism of action, and additional optimization of dosing and investigation of adverse effects are warranted. In addition, further exploration of the factors that lead to a dramatic response in some individuals, and no response in others is indicated in a much larger patient cohort. Findings from such a study may be applicable both to dogs and to humans with chronic spinal cord injury.

## Supporting Information

S1 Fig **Owner questionnaire and DNI categories.** Left: Questionnaire is designed for owner to assess overall function of their dog at home; owners are asked to check yes or no and then to identify specific response; TL-SCI: thoracolumbar spinal cord injury; Right: DNI scale; questionnaires are graded by a blinded clinician using the D (deterioration), N (not changed) and I (improvement) scale.
(TIF)

S1 Table **Modified open field score of gait.**
(DOCX)

S1 Dataset **Primary and secondary outcome data for each individual dog.** Grey hatching indicates t-butyl doses that were sub-therapeutic. This data and data from the preceding 2 weeks were excluded from analysis.
(PDF)

S1 Text **Full description of the statistical model developed to analyze data.**
(DOCX)

S1 Video **Videotape of dog 6 walking unsupported on the treadmill as he moved through each phase of the protocol.** The phase of treatment is listed in the bottom right hand corner of the video.
(MP4)
