# Supplementary figures and images for: Potassium Channel Antagonists 4-Aminopyridine and the T-Butyl Carbamate Derivative of 4-Aminopyridine Improve Hind Limb Function in Chronically Non-Ambulatory Dogs; A Blinded, Placebo-Controlled Trial
Source: PLoS One. 2014 Dec 31;9(12):e116139. doi: 10.1371/journal.pone.0116139 (PMC4281252; doi:10.1371/journal.pone.0116139)

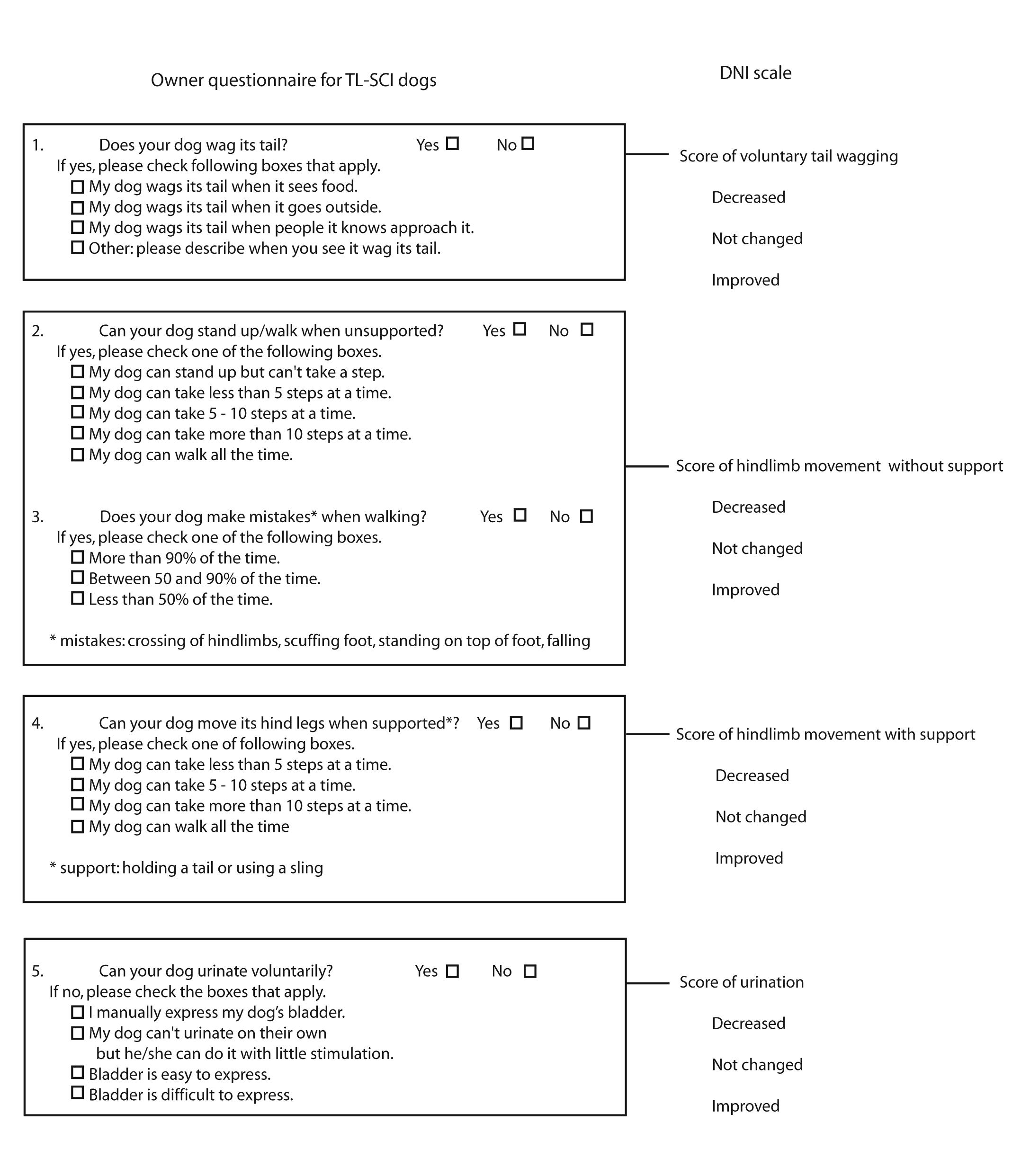

Supplement: S1 Fig — Owner questionnaire and DNI categories. Left: Questionnaire is designed for owner to assess overall function of their dog at home; owners are asked to check yes or no and then to identify specific response; TL-SCI: thoracolumbar spinal cord injury; Right: DNI scale; questionnaires are graded by a blinded clinician using the D (deterioration), N (not changed) and I (improvement) scale. (TIF) [file pone.0116139.s001.tif]
