# Supplementary material for: Potassium Channel Antagonists 4-Aminopyridine and the T-Butyl Carbamate Derivative of 4-Aminopyridine Improve Hind Limb Function in Chronically Non-Ambulatory Dogs; A Blinded, Placebo-Controlled Trial
Source: PLoS One. 2014 Dec 31;9(12):e116139. doi: 10.1371/journal.pone.0116139 (PMC4281252; doi:10.1371/journal.pone.0116139)
Supplement: S1 Table — Modified open field score of gait. (DOCX) [file pone.0116139.s002.docx]

**Table S1. Modified open field score of gait from [44].**

| Score | Description |
| --- | --- |
| 0 | Paraplegic |
| 1 | Paraplegic with voluntary tail wag. |
| 2 | Minimal non-weight bearing protraction of pelvic limb (movement of 1 joint) |
| 3 | Non-weight bearing protraction of pelvic limb with > 1 joint involved < 50% of time. |
| 4 | Non-weight bearing protraction of pelvic limb with > 1 joint involved > 50% of time. |
| 5 | Weight bearing protraction of pelvic limb < 10% of time. |
| 6 | Weight bearing protraction of pelvic limb 10-50% of time. |
| 7 | Weight bearing protraction of pelvic limb > 50% of time. |
| 8 | Weight bearing protraction 100% of time with reduced strength of pelvic limb. Mistakes > 90% of time (crossing of pelvic limbs, scuffing foot on protraction, standing on dorsum oh foot, falling). |
| 9 | Weight bearing protraction 100% of time with reduced strength of pelvic limb. Mistakes 50-90% of time. |
| 10 | Weight bearing protraction 100% of time with reduced strength of pelvic limb. Mistakes < 50% of time. |
| 11 | Ataxic pelvic limb gait with normal strength but mistakes > 50% of time (lack of coordination with thoracic limb, crossing of pelvic limbs, skipping steps, bunny hopping, scuffing foot on protraction, standing on dorsum of foot). |
| 12 | Ataxic pelvic limb gait with normal strength but mistakes < 50% of time. |
| 13 | Normal pelvic limb gait. |
