# Supplementary material for: Potassium Channel Antagonists 4-Aminopyridine and the T-Butyl Carbamate Derivative of 4-Aminopyridine Improve Hind Limb Function in Chronically Non-Ambulatory Dogs; A Blinded, Placebo-Controlled Trial
Source: PLoS One. 2014 Dec 31;9(12):e116139. doi: 10.1371/journal.pone.0116139 (PMC4281252; doi:10.1371/journal.pone.0116139)
Supplement: S1 Dataset — Primary and secondary outcome data for each individual dog. Grey hatching indicates t-butyl doses that were sub-therapeutic. This data and data from the preceding 2 weeks were excluded from analysis. (PDF) [file pone.0116139.s003.pdf]

Dog NO.	Video ID	Week	Treatment	Unsupported		Supported		OFS	DNI score			
				SS	RI	SS	RI		Tail function	HL function wo support	HL function w/support	Bladder function
1	A82	1	placebo	0.00	0.00	6.00	0.00	3	U	U	U	U
1	A62	2	placebo	0.00	0.00	2.00	0.00	3	U	U	U	U
1	A47	3	TBOC lo	0.00	0.00	7.00	0.00	3	U	U	U	U
1	A74	4	TBOC lo	0.00	0.00	17.00	0.00	4	U	U	U	U
1	A32	5	washout	0.00	0.00	15.00	0.00	5	U	U	U	U
1	A87	6	washout	0.00	0.00	4.00	0.00	5	U	U	U	U
1	A21	7	4AP	0.00	0.00	37.50	5.87	4.5	U	U	U	U
1	A41	8	4AP	0.00	0.00	37.50	7.39	4.5	U	U	U	U
1	A43	9	placebo	0.00	0.00	4.00	0.00	4	U	U	U	U
1	A27	10	placebo	0.00	0.00	9.50	0.00	3.5	U	U	U	U
1	A93	11	TBOC	0.00	0.00	37.00	2.92	5	U	U	I	U
1	A61	12	TBOC	0.00	0.00	36.00	4.17	5	U	U	U	U
2	B29	1	placebo	0.00	0.00	0.00	0.00	1.5	U	U	U	U
2	B3	2	placebo	0.00	0.00	0.00	0.00	1.5	U	U	U	U
2	B73	3	4AP	0.00	0.00	0.00	0.00	1.5	U	U	U	U
2	B80	4	4AP	0.00	0.00	0.00	0.00	3	U	U	U	U
2	B67	5	washout	0.00	0.00	0.00	0.00	3.5	U	U	U	U
2	B22	6	washout	0.00	0.00	0.00	0.00	1.5	U	U	U	U
2	B91	7	TBOC lo	0.00	0.00	0.00	0.00	1.5	U	U	U	U
2	B8080	8	TBOC lo	0.00	0.00	0.00	0.00	3.5	U	U	U	U
2	B24	9	placebo	0.00	0.00	0.00	0.00	1.5	U	U	U	U
2	B27	10	placebo	0.00	0.00	0.00	0.00	3	I	U	I	U
2	B61	11	TBOC	0.00	0.00	37.00	0.00	4.5	I	U	I	U
2	B99	12	TBOC	0.00	0.00	38.50	0.00	5	U	U	I	U
3	C14	1	placebo	0.00	0.00	22.00	6.56	4	U	U	U	U
3	C15	2	placebo	0.00	0.00	0.00	0.00	3	U	U	U	U
3	C68	3	TBOC lo	0.00	0.00	2.00	0.00	3	U	U	U	U
3	C93	4	TBOC lo	0.00	0.00	0.00	0.00	3	U	U	U	U
3	C57	5	washout	0.00	0.00	1.00	0.00	4	U	U	U	U
3	C46	6	washout	0.00	0.00	20.50	0.00	5	U	U	U	U
3	C11	7	4AP	0.00	0.00	46.50	16.41	5	U	U	I	U
3	C88	8	4AP	0.00	0.00	20.00	10.00	5	U	U	I	U
3	C13	9	placebo	0.00	0.00	0.50	0.00	4	U	U	I	U
3	C67	10	placebo	0.00	0.00	29.00	10.82	5	U	U	U	U
4	D9	1	placebo	0.00	0.00	0.00	0.00	1.5	U	U	U	U
4	D38	2	placebo	0.00	0.00	0.00	0.00	1.5	U	U	U	U
4	D1	3	TBOC lo	0.00	0.00	0.00	0.00	3	U	U	U	I
4	D95	4	TBOC lo	0.00	0.00	0.00	0.00	3	U	U	U	U
4	D98	5	washout	0.00	0.00	0.00	0.00	1	U	U	U	D
4	D33	6	washout	0.00	0.00	0.00	0.00	1.5	U	U	U	U
4	D72	7	4AP	0.00	0.00	0.00	0.00	3.5	U	U	U	I
4	D16	8	4AP	0.00	0.00	0.00	0.00	3	I	U	U	I
4	D17	9	placebo	0.00	0.00	0.00	0.00	1.5	U	U	U	I
4	D54	10	placebo	0.00	0.00	0.00	0.00	0	U	U	U	I
5	F32	1	placebo	0.00	0.00	38.00	11.57	5.5	U	U	U	U
5	F69	2	placebo	0.00	0.00	57.50	20.48	6	U	I	I	U
5	F87	3	4AP	0.00	0.00	44.00	26.39	5.5	U	U	U	U
5	F81	4	4AP	1.50	0.00	56.00	14.37	6.5	U	U	I	U
5	F28	5	washout	0.00	0.00	54.00	18.18	6	U	U	I	U
5	F14	6	washout	0.00	0.00	33.00	20.74	5	U	U	I	U
5	F06	7	TBOC lo	0.00	0.00	38.00	5.80	5	U	U	I	U
5	F37	8	TBOC lo	0.00	0.00	61.00	12.42	6	U	U	I	U
5	F52	9	placebo	0.00	0.00	37.00	8.80	5	U	U	I	U
5	F59	10	placebo	0.00	0.00	34.50	11.90	5.5	U	U	I	U
5	F42	11	TBOC	0.00	0.00	52.50	17.06	6	U	U	I	U
5	F09	12	TBOC	1.00	0.00	52.50	11.51	6	U	I	I	U
6	G73	1	placebo	0.00	0.00	19.00	3.36	4	U	U	U	U
6	G35	2	placebo	0.00	0.00	16.00	1.64	5	U	U	I	U

6	G97	3	TBOC lo	5.50	0.00	8.00	0.00	5	U	I	U	U
6	G77	4	TBOC lo	0.00	0.00	6.00	0.00	5	U	I	I	U
6	G32	5	washout	7.50	3.74	25.00	8.06	5.5	U	I	U	U
6	G18	6	washout	7.00	1.83	39.50	2.87	6	U	I	I	U
6	G37	7	4AP	6.67	0.00	55.50	29.58	7	U	I	I	U
6	G40	8	4AP	32.85	4.40	48.50	17.52	6	U	I	I	U
6	G45	9	placebo	12.00	3.51	20.50	8.25	5	U	I	I	U
6	G33	10	placebo	11.50	5.22	29.00	3.10	6	U	I	I	U
6	G01	11	TBOC	28.50	15.69	35.00	4.46	5	U	I	I	U
6	G99	12	TBOC	41.00	14.15	42.50	9.83	5.5	U	I	I	U
7	H21	1	placebo	0.00	0.00	17.00	0.00	5	U	U	U	U
7	H18	2	placebo	0.00	0.00	23.50	15.32	5	N/A	N/A	N/A	N/A
7	H50	3	TBOC lo	0.00	0.00	9.00	0.00	5	U	U	U	U
7	H86	4	TBOC lo	0.00	0.00	13.00	7.08	5	U	U	I	U
7	H29	5	washout	0.00	0.00	15.00	6.96	5	U	U	I	U
7	H62	6	washout	0.00	0.00	11.00	3.57	5	U	U	U	U
7	H40	7	4AP	0.00	0.00	42.50	9.83	5	U	U	U	U
7	H45	8	4AP	0.00	0.00	40.00	19.83	5	U	U	I	U
7	H42	9	placebo	0.00	0.00	13.00	1.71	5	U	U	U	U
7	H22	10	placebo	0.00	0.00	6.50	1.89	5	U	U	I	U
7	H66	11	TBOC	0.00	0.00	5.50	0.00	5	U	U	I	U
7	H15	12	TBOC	0.00	0.00	13.00	0.00	5	U	U	I	U
8	I03	1	placebo	N/A	N/A	N/A	N/A	5	U	U	U	U
8	I32	2	placebo	0.00	0.00	46.50	20.47	5	U	U	I	U
8	I01	3	4AP	0.00	0.00	67.00	9.58	5	U	U	I	U
8	I73	4	4AP	0.00	0.00	49.00	9.44	5	U	U	I	U
8	I30	5	washout	0.00	0.00	44.00	12.50	5	U	U	I	U
8	I23	6	washout	0.00	0.00	25.00	11.20	5	U	U	I	U
8	I91	7	TBOC	0.00	0.00	50.50	15.91	5	U	U	I	U
8	I87	8	TBOC	0.00	0.00	50.00	6.65	5	U	U	I	U
8	I06	9	placebo	0.00	0.00	45.00	15.22	5.5	U	U	I	U
8	I49	10	placebo	0.00	0.00	45.00	20.84	5	U	U	I	U
9	J67	1	placebo	0.00	0.00	42.00	9.80	5	U	U	U	U
9	J79	2	placebo	0.00	0.00	42.00	9.86	5	U	U	U	U
9	J71	3	4AP	0.00	0.00	45.50	23.38	6	U	I	I	U
9	J32	4	4AP	0.00	0.00	32.00	3.03	N/A	U	I	I	U
9	J69	5	washout	0.00	0.00	35.00	1.53	N/A	U	U	U	U
9	J19	6	washout	0.00	0.00	25.50	3.25	5	U	U	U	U
9	J16	7	TBOC	0.00	0.00	41.50	11.34	5	U	U	U	U
9	J84	8	TBOC	0.00	0.00	23.00	3.28	5	U	I	I	U
9	J62	9	placebo	0.00	0.00	37.00	11.69	5	U	U	U	U
9	J21	10	placebo	0.00	0.00	39.00	4.38	5.5	U	U	U	U
10	K52_1	1	placebo	0.00	0.00	41.50	12.60	5	U	U	U	U
10	K30	2	placebo	0.00	0.00	22.00	0.00	5	U	I	I	U
10	K01	3	TBOC	0.00	0.00	23.00	10.74	5	U	U	U	U
10	K59	4	TBOC	0.00	0.00	50.00	18.67	5	N/A	N/A	N/A	N/A
10	K56	5	washout	0.00	0.00	42.00	22.54	5	U	U	U	U
10	K38	6	washout	0.00	0.00	24.00	3.23	5	U	U	U	U
10	K19	7	4AP	0.00	0.00	40.00	11.43	5	U	U	I	U
10	K33	8	4AP	0.00	0.00	54.00	12.99	5	U	U	I	U
10	K52_2	9	placebo	0.00	0.00	28.00	21.88	5	U	U	I	U
10	K09	10	placebo	0.00	0.00	22.00	8.13	5	U	U	I	U
11	L45	1	placebo	0.00	0.00	0.00	0.00	0	U	U	U	U
11	L84	2	placebo	0.00	0.00	0.00	0.00	0	U	U	I	U
11	L13	3	4AP	0.00	0.00	0.00	0.00	0	U	U	U	U
11	L91	4	4AP	0.00	0.00	0.00	0.00	N/A	N/A	N/A	N/A	N/A
12	M54	1	placebo	0.00	0.00	0.00	0.00	3.5	U	U	U	U
12	M68	2	placebo	0.00	0.00	0.00	0.00	4	U	U	U	U
12	M80	3	TBOC	0.00	0.00	0.00	0.00	4.5	I	U	U	U
12	M60	4	TBOC	0.00	0.00	0.00	0.00	4.5	U	U	U	U
13	N67	1	placebo	0.00	0.00	0.00	0.00	2	U	U	U	U
13	N93	2	placebo	0.00	0.00	0.00	0.00	2.5	U	U	U	U
13	N84	3	TBOC	0.00	0.00	0.00	0.00	3	U	U	U	U
13	N45	4	TBOC	0.00	0.00	0.00	0.00	2.5	U	U	I	U
13	N13	5	washout	0.00	0.00	0.00	0.00	1	D	U	I	U
13	N91	6	washout	0.00	0.00	0.00	0.00	3	U	U	I	U

14	O32	1	placebo	1.00	0.00	30.00	16.75	4	U	U	U	U
14	O92	2	placebo	0.00	0.00	24.48	8.16	4	U	U	U	U
14	O68	3	4AP	0.00	0.00	50.50	9.34	5	U	I	I	U
14	O25	4	4AP	0.00	0.00	63.00	18.41	5	U	I	I	U
14	O33	5	washout	0.00	0.00	19.38	4.21	4	U	I	I	U
14	O53	6	washout	0.00	0.00	38.00	14.49	4	U	I	I	U
14	O23	7	TBOC	0.00	0.00	41.00	0.00	4	U	I	I	U
14	O89	8	TBOC	0.00	0.00	20.00	3.33	4	U	I	I	U
14	O85	9	placebo	0.00	0.00	71.50	37.34	5.5	U	I	I	U
14	O41	10	placebo	0.00	0.00	38.50	4.35	4	U	I	I	U
15	P88	1	placebo	0.00	0.00	0.00	0.00	1	U	U	U	U
15	P91	2	placebo	0.00	0.00	0.00	0.00	1	U	U	U	U
15	P46	3	4AP	0.00	0.00	50.00	58.67	4	U	U	I	U
15	P22	4	4AP	0.00	0.00	39.00	28.78	4	U	U	I	U
15	P50	5	washout	0.00	0.00	4.00	0.00	1	U	U	I	U
15	P10	6	washout	0.00	0.00	0.00	0.00	1	U	U	U	U
15	P33-1	7	TBOC	0.00	0.00	30.00	36.92	4	U	U	U	U
15	P33-2	8	TBOC	0.00	0.00	0.00	0.00	1	U	U	U	U
15	P95	9	placebo	0.00	0.00	10.00	0.00	3	U	U	U	U
15	P01	10	placebo	0.00	0.00	34.00	5.97	3	U	U	U	U
16	Q58	1	placebo	0.00	0.00	0.00	0.00	N/A	U	U	U	U
16	Q62	2	placebo	0.00	0.00	0.00	0.00	0	U	U	U	U
16	Q20	3	TBOC	0.00	0.00	0.00	0.00	3	I	U	U	U
16	Q97	4	TBOC	0.00	0.00	0.00	0.00	0	I	U	U	U
16	Q79	5	washout	0.00	0.00	0.00	0.00	0	U	U	U	U
16	Q84	6	washout	0.00	0.00	0.00	0.00	0	U	U	U	U
16	Q44	7	4AP	0.00	0.00	7.00	3.74	4	I	U	U	U
16	Q28	8	4AP	0.00	0.00	57.00	20.38	4	I	U	U	U
16	Q04	9	placebo	0.00	0.00	0.00	0.00	3	U	U	U	U
16	Q47	10	placebo	0.00	0.00	2.00	0.00	3	U	U	U	U
17	R75	1	placebo	0.00	0.00	63.00	9.82	4	U	U	U	U
17	R32	2	placebo	0.00	0.00	44.00	8.33	5	U	U	U	U
17	R29	3	TBOC	48.00	8.11	53.00	20.92	6	U	U	U	U
17	R67	4	TBOC	41.00	5.67	49.00	10.74	5	U	U	U	U
17	R27	5	washout	29.00	3.10	47.00	13.61	6	U	U	U	U
17	R28	6	washout	51.00	21.19	51.00	15.89	6	U	U	U	U
17	R39	7	4AP	50.00	13.33	51.00	10.60	6	I	I	U	U
17	R69	8	4AP	40.00	8.57	47.00	13.61	6	I	I	I	U
17	R94	9	placebo	35.00	11.85	32.00	3.03	5	U	U	U	U
17	R93	10	placebo	23.08	6.25	44.00	13.89	5	U	U	U	U
18	S71	1	placebo	0.00	0.00	35.00	17.78	5	I	I	I	U
18	S79	2	placebo	0.00	0.00	33.00	12.03	5	U	U	I	U
18	S52	3	TBOC	0.00	0.00	57.00	17.83	5	U	U	U	U
18	S61	4	TBOC	0.00	0.00	38.00	2.90	5	U	I	U	U
18	S27	5	washout	0.00	0.00	28.00	6.25	5	I	I	I	U
18	S51	6	washout	0.00	0.00	50.00	18.67	5	I	I	U	U
18	S80	7	4AP	39.00	20.14	53.00	13.07	6	I	I	I	I
18	S46	8	4AP	24.00	16.13	56.00	23.08	6	I	I	I	I
18	S66	9	placebo	0.00	0.00	52.00	10.53	5	I	I	I	I
18	S04	10	placebo	0.00	0.00	34.00	8.96	5	I	I	I	I
19	T39	1	placebo	0.00	0.00	11.00	1.82	5	U	U	U	U
19	T41	2	placebo	0.00	0.00	5.50	0.00	4	U	U	U	U
19	T32	3	4AP	0.00	0.00	50.00	18.71	5	U	U	I	U
19	T84	4	4AP	0.00	0.00	27.00	0.00	5.5	U	U	U	U
19	T08	5	washout	0.00	0.00	36.50	7.32	5.5	U	U	U	U
19	T13	6	washout	0.00	0.00	48.50	18.80	5	U	U	U	U
