# Supplementary material for: Potassium Channel Antagonists 4-Aminopyridine and the T-Butyl Carbamate Derivative of 4-Aminopyridine Improve Hind Limb Function in Chronically Non-Ambulatory Dogs; A Blinded, Placebo-Controlled Trial
Source: PLoS One. 2014 Dec 31;9(12):e116139. doi: 10.1371/journal.pone.0116139 (PMC4281252; doi:10.1371/journal.pone.0116139)
Supplement: S1 Text — Full description of the statistical model developed to analyze data. (DOCX) [file pone.0116139.s004.docx]

**Text S1.** Statistical Analysis: detailed description of the statistical model used to analyze the data and the resulting data analysis.

A statistical model was designed to analyze the data in this trial that took into consideration the attrition of 6 patients at different stages in the trial by examining treatment effect in each individual, and that examined the possibility of a carryover effect of treatment over time, by comparing the periods during which there was no treatment with each other. The model compares the outcome measure scores in the 2 weeks pre treatment with the treatment weeks in individual patients, and compares the outcome scores during treatment with 4AP and t-butyl. A detailed mathematical description of the model development is given below.

The treatment assignments for each of the n = 19 subjects are displayed on the right-hand side of Figure 1. It can be seen that 13 subjects experienced cross-over and received both treatments (4 t-butyl then 4-AP, and 9 4-AP then t-butyl); 4 subjects received 4-AP only and 2 subjects received t-butyl only. We consider the effect of treatment on seven different outcomes that are listed in Table 2. To account for individual subject effects we consider all outcomes under treatment relative to the outcome under the control period immediately preceding treatment. Thus, it is convenient use a time-scale of two-week blocks (block 1:week 1/2; block 2: week 3/4; block 3:week 5/6; block 4: week 7/8; block 5: week 9/10; block 6: week 11/12).

Let A *_i,j_* denote the treatment received by subject *i* during block *j*,

e.g., A*1,2* is the treatment received by subject 1 during weeks 3 and 4.

Let M ^k^ *_i,j_* denote the average of weekly measurements of outcome *k* measured on subject *i* during two-week block *j*,

e.g., if the fifth outcome is a subject’s OFS score, then M^5^*2,4* denotes the average of the OFS scores collected at weeks 7 and 8 for subject 2.

Let Z *_i,j_* denote an indicator that subject *i* has completed a course of t-butyl prior to block j and let W *i,j* denote an indicator that subject *i* has completed a course of 4-AP prior to block *j*.

We assume a generative model of the form

*M^k^_i,j_* = µ*_i_* + *a^k^*_0_ +*a^k^*_1_*1_Ai,j_* = t-butyl + *a^k^*_2_*1_Ai,j_* = 4-AP+ *r^k^_1,i_* Z*_i,j_ +* *r^k^_2,i_W_i,j_ + ∆^k^_i,I_* (1)

Where 1_u_ is equal to one if u is true and zero otherwise, *a^k^*_0,_ *a^k^*_1,_ *a^k^*_2_ are unknown parameters common to all subjects, µ*_i,_ r^k^_1,i,_ r^k^_2,i_* are unknown subject-specific parameters, and (*∆^k^_i,1,_ ∆^k^_i,2,_ ∆^k^_i,3,_ ∆^k^_i,4,_ ∆^k^_i,5,_ ∆^k^_i,6_)* are multivariate normal errors with mean zero. This model allows for a subject-specific mean (µ*_i_*) and subject-specific carryover effect of past treatments (*r^k^_1,i,_ r^k^_2,i_*).

The analysis model we derive from (1) does not depend on subject-specific effects.

One potential concern with (1) is that the treatment effect might dissipate slowly over time thereby violating the additive subject-specific carryover effect. Under such a dissipating carryover effect we would expect a difference in between the outcomes in the pre-treatment and post-treatment washout periods.

Let *S^k^_i_* = sgn(*M^k^_i,1_ - M^k^_i,3_*) where sgn(u) is 1 if u > 0, -1 if u < 0, and 0 otherwise. Let *I* denote the set of subjects receiving an active treatment in weeks 3-4. If there is no carryover effect then we would expect an equal number of 1's and -1's in the set {*S^k^_i_*  , *i* ∈ *I* }. Formally, we assume {*S^k^_i_*  , *i* ∈ *I* } are independent and identically distributed and we test the null hypothesis H_0_ : *P*(*S^k^_1_* =1) = *P*(*S^k^_1_* = -1) for each outcome *k* using a Χ^2^ test. Below table shows the test statistics and corresponding p-values; there is no evidence of a carryover effect in any of the outcomes under consideration.

| Outcome | Χ^2^ | p-values |
| --- | --- | --- |
| OFS | 1.8 | 0.18 |
| SS+ | 0.09 | 0.76 |
| RI+ | 0.11 | 0.74 |
| Tail function DNI | 0.0 | 1.0 |
| Bladder function DNI | 0.0 | 1.0 |
| Hindlimb function without support DNI | 0.14 | 0.71 |
| Hindlimb function with support DNI | 1.3 | 0.26 |

Table legend: Chi-square test comparing outcomes in weeks 1-2 with outcomes in weeks 5-6 using data from subjects receiving active treatment in weeks 3-4. Note *OFS: open field score; SS+:stepping score with support; RI+: regularity index with support; DNI: owners’ questionnaire score

For each (*I, j, k*) triple such that *A_i,j_* is either t-butyl or 4-AP, and outcome *k* is measured on subject *i* in blocks *j* and j -1, define *Y ^k^_i,j_ = M^k^_i,j_ – M^k^_i,j_*_-1_. Model (1) implies

*Y^k^_i,j_* = *ß^k^*_0_ +*ß^k^*_1_ *A_i,j_* +*e^k^_i,j_* (2)

where *ß^k^*_0_ , *ß^k^*_1_ are unknown parameters, *e^k^_i,j_* are mean zero normal errors that are assumed to be independent across subjects and satisfy Var(*e^k^_i,j_*) = *∆^2^_k_* and Cov (*e^k^_i,j_* , *e^k^_i,l_* )= *t_k_* if *l* ≠ *j*. We code *A_i,j_* to take values in {-1, 1} with 1 denoting 4-AP and -1 denoting t-butyl; with this coding *ß^k^*_0_ represents the main effect of treatment relative to placebo and *ß^k^*_1_ represents the relative difference between 4-AP and t-butyl.

Let *J _i_* denote the blocks in which subject *i* received either 4-AP or t-butyl. The data used to estimate the parameters in (2) are {(*Y^k^_i,j_*, *A_i,j_*),*j* ∈ *J _i_ , i=*1, …,n) ; thus, subjects that complete one course of active treatment contribute one observation to the data and subjects completing two courses of active treatment contribute two observations. Any unique characteristics of patients dropping out of the study are assumed to be captured in the subject-specific mean and carry-over effects in (1). We estimate the parameters *ß^k^*_0_ and *ß^k^*_1_ using maximum likelihood. The estimated parameters are given in Table below.

| Outcome | *ß^k^*_0_ | (p-values) | *ß^k^*_1_ | (p-values) | Ave. Imp. |
| --- | --- | --- | --- | --- | --- |
| OFS | 0.62 | (0.005) | 0.15 | (0.31) | 0.66 |
| SS+ | 13.6 | (<0.0001) | 1.9 | (0.51) | 13.4 |
| RI+ | 4.7 | (0.028) | 2.2 | (0.13) | 4.9 |
| Tail function DNI | 0.055 | (0.29) | -0.002 | (0.94) | 0.063 |
| Bladder function DNI | 0.059 | (0.18) | 0.059 | (0.18) | 0.063 |
| Hindlimb function without support DNI | 0.25 | (0.076) | 0.061 | (0.54) | 0.27 |
| Hindlimb function with support DNI | 0.17 | (0.035) | 0.17 | (0.13) | 0.17 |

Table legend: Estimated coefficients *ß^k^*_0,_ *ß^k^*_1_ for each of the outcome; treatments are coded to take values in {-1, 1} with 4-AP coded as 1 and t-butyl as -1. Four of the seven outcomes show significant main effects (*ß^k^*_0_ ) at the 0.05 level (not adjusted for multiplicity). There was no significant difference between t-butyl and 4-AP (*ß^k^*_1_) at the 0.05 level in any of the outcomes under consideration. The last column shows the average improvement over placebo computed as the average of *Y^k^_i,j_* over all (*i, j)* for which *Y^k^_i,j_* is defined.

The main effect of treatment was significant at the 0.05 level for four of the seven outcomes under consideration; adjusting for multiplicity via the Bonferroni correction a 0.05 significance level becomes 0.05/7 = 0.007 at which the main effect of treatment was significant in two of the seven outcomes. There was no significant difference between 4-AP and t-butyl at the 0.05 level for any of the outcomes.
